# Thoracolumbar Extramedullary Myxopapillary Ependymoma: A Rare Case

**DOI:** 10.7759/cureus.30402

**Published:** 2022-10-17

**Authors:** Radha A Channawar, Swapnil Date, Sanjay V Deshpande, Ventaktesh Dasari, Prashanth Balusani

**Affiliations:** 1 Department of Orthopaedics and Traumatology, Jawaharlal Nehru Medical College, Datta Meghe Institute of Medical Sciences, Wardha, IND; 2 Department of Orthopaedics and Traumatology, Jawaharlal Nehru Medical College, Datta Meghe Institute of Medical Science, Wardha, IND

**Keywords:** cauda equina, paraplegia, lower back pain, myxopapillary ependymoma, ependymoma

## Abstract

An intradural extramedullary myxopapillary ependymoma is a rare spinal cord tumor found in the thoracolumbar region of the spine. In this case report, we describe this case and review the various aspects of ependymomas. There are different types of ependymomas based on their location and histopathological appearance. Myxopapillary ependymoma is a non-invasive, slow-growing benign tumor that can present as a simple complaint of lower back pain to a severe stage like paraplegia.

## Introduction

Ependymoma is a benign tumor of ependymal cells of the spinal cord and central nervous system [1,2]. Ependymal cells are present in the lining of the central canal of the spinal cord. These have diverse functions ranging from stem cell potential to astrocyte production after any injury to the spinal cord [2,3]. Ependymomas have a bimodal distribution in occurrence. In children, it is largely found in the brain, whereas in adults, it is located in the spinal cord [2,4]. Ependymomas are divided into the following grades by the WHO. These tumors cannot be staged according to the conventional TNM (T - tumor, N - nodes, M - metastasis) staging as they do not invade any adjacent structures frequently [2,4,5]. Myxopapillary ependymomas are thin on-ground tumors located intradural extramedullary. They are more likely found in the lumbosacral area of the spine. Here, myxopapillary implies the histopathological appearance of scattered clear mucin pools in the stroma and the round to elongated cells arranged radially [4,6,7]. They are usually found in parts of the spinal cord like - conus medullaris, cauda equina, and filum terminale. In contrast, the occurrence in the cervicothoracic, lateral ventricles, and brain parenchyma is rare [2,4,6,8]. The best diagnostic modalities are MRI and CT scans of the spine, and histopathology can further refine the diagnosis [6]. Appropriate consent was taken before presenting this case report.

## Case presentation

History of present illness

A 67-year-old female presented with complaints of pain in the lower back for two years, and weakness in both lower limbs due to which she was unable to walk. She lost her bladder and bowel control and developed dribbling of urine. The pain was insidious in onset and gradually progressed. Severity had increased in the last month. It was hindering her daily activities. Pain radiated to both feet. The pain was constant throughout the day, aggravated by movements, and reduced by medications and rest. The pain was also associated with a tingling sensation in both lower limbs. There was no diurnal variation. There was no history of heavy weight lifting or trauma. The bladder and bowel involvement was present.

Examination

The patient was lying on a bed. Spinal curvature was normal, and the local temperature was not raised. Bony tenderness was present over T11-L1, and paraspinal muscle spasm was present. On the Straight leg raising test, the patient felt pain on the left side at 20° and on the right side at 30°. The Lasegue sign was positive. Sensations were intact. All the deep tendon reflexes were elicited. Higher functions and cranial nerves were intact. Muscles were hypotonic. Planters were non-responsive. Wasting of muscles of lower limbs was present. Table 1 represents the result of the examination of power in the muscles of the lower limbs of the patient. Examination findings imply that probably the anterior fibers were spared therefore, the sensory system is spared. Also, the motor fibers are more sensitive therefore, their manifestations develop prominently.

**Table 1:** Power of lower limb muscles 0/V represents grade zero power that means no muscle contraction was seen

Joint	Movement	Left leg	Right leg
Hip	Flexion	0/V	0/V
	Extension	0/V	0/V
	Adduction	0/V	0/V
	Abduction	0/V	0/V
Knee	Flexion	0/V	0/V
	Extension	0/V	0/V
Ankle	Dorsiflexion	0/V	0/V
	Plantar flexion	0/V	0/V
	Extensor hallucis longus / extensor digitorum longus	0/V	0/V
	Flexor hallucis longus / flexor digitorum longus	0/V	0/V

Investigations

**Figure 1:**
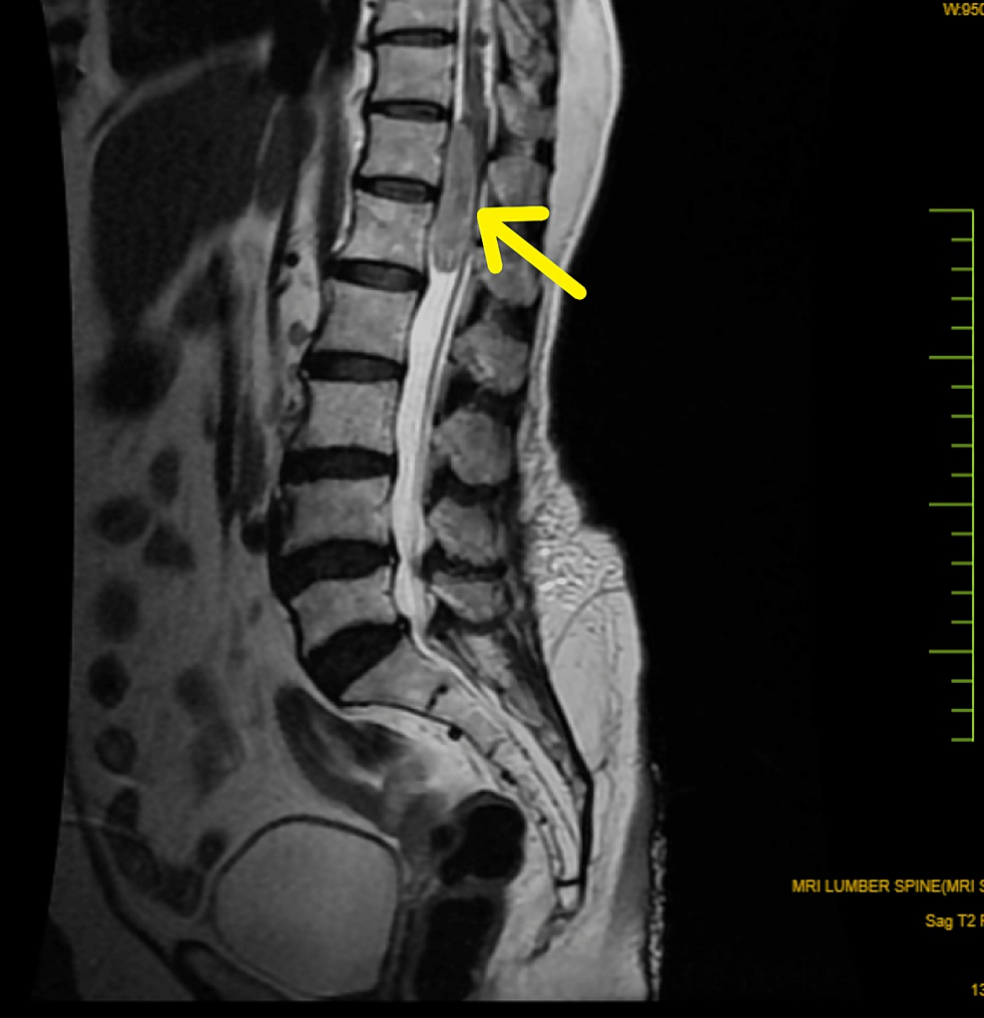
T2-Weighted MRI (sagittal section) The image shows a well-circumscribed enhancing lesion extending from the upper border of T12 to the lower border of L1. Lesion is intradural extramedullary.

**Figure 2:**
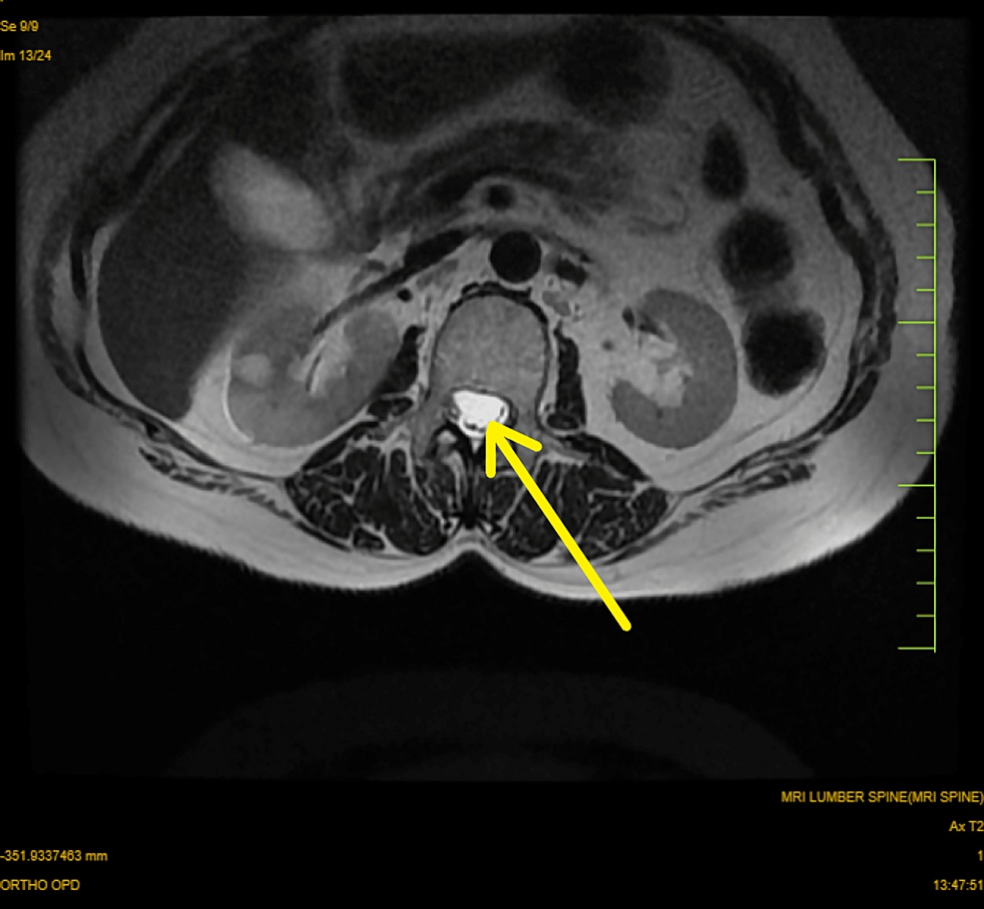
MRI (transverse section) The yellow arrow is showing the tumor

The patient underwent an MRI (Magnetic Resonance Imaging) of the spine, where a well defined longitudinally oriented, altered signal intensity lesion was noted in the intradural, extramedullary compartment extending from the upper border of D12 to the lower border of the L1 vertebra (in front of conus medularis), showing hyperintensity on T2 weighted (T2W) MRI and showing post-contrast enhancement. The lesion was displacing the cord posteriorly. Figure 1 depicts the tumor in the T2W MRI of the LS spine and Figure 2 depicts the tumor on the transverse section MRI.

Diagnosis

A myxopapillary ependymoma (WHO grade I) over T12-L1 with paraplegia was diagnosed.

Treatment 

**Figure 3:**
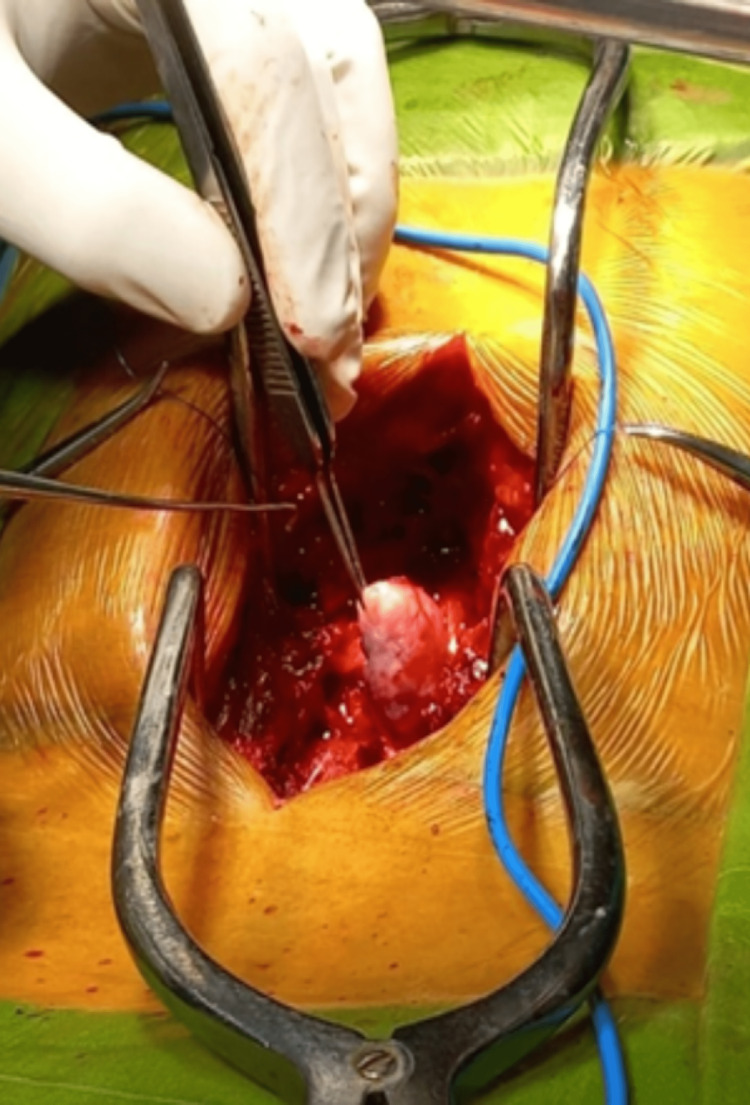
Visualization of the Myxopapillary Ependymoma of the spine

**Figure 4:**
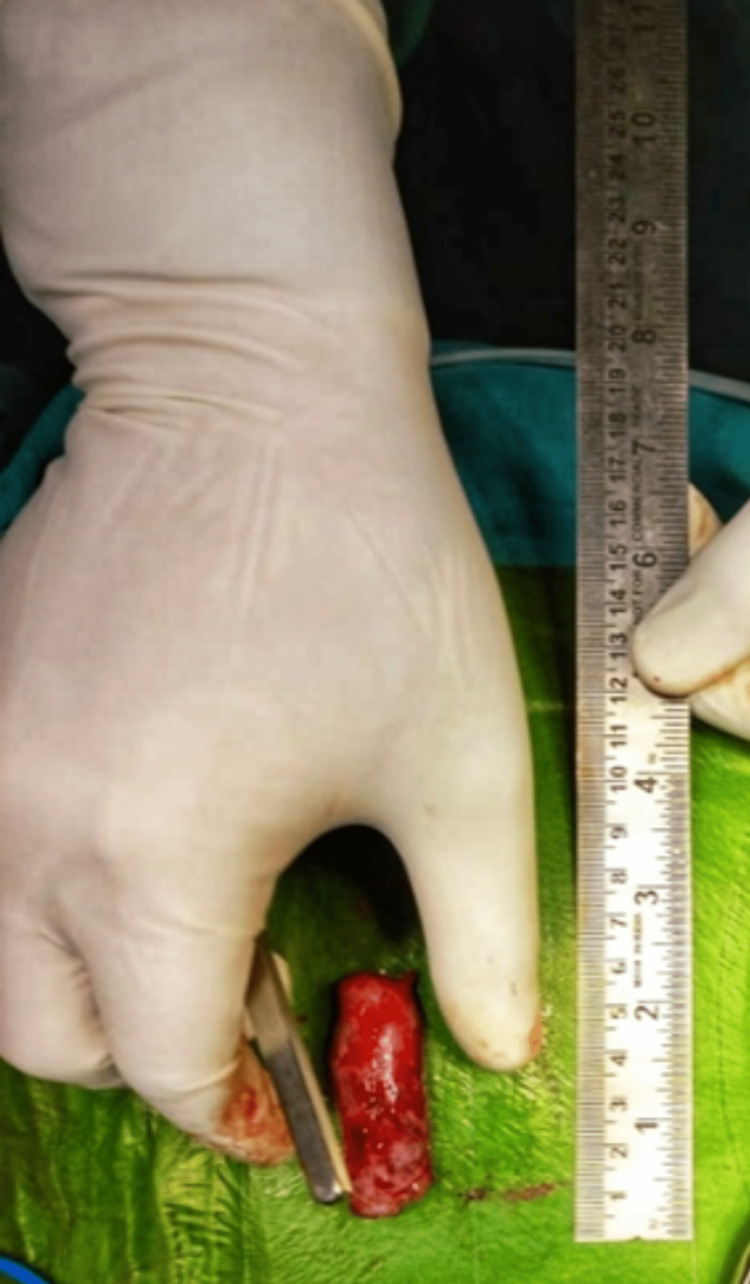
Excised single, tubular Myxopapillary Ependymoma of the spine measuring around 5 x 1.5 x 0.8 cm

Posterior decompression and excision of tumor at T12-L1 level for space-occupying lesion over T12 -L1 done under general anesthesia. The patient was advised post-operative radiotherapy to prevent a recurrence. Further, antibiotic coverage and pain relief were managed. Figures 3-4 show the intraoperative visualization and measurements of the tumor respectively.

Histopathological findings

**Figure 5:**
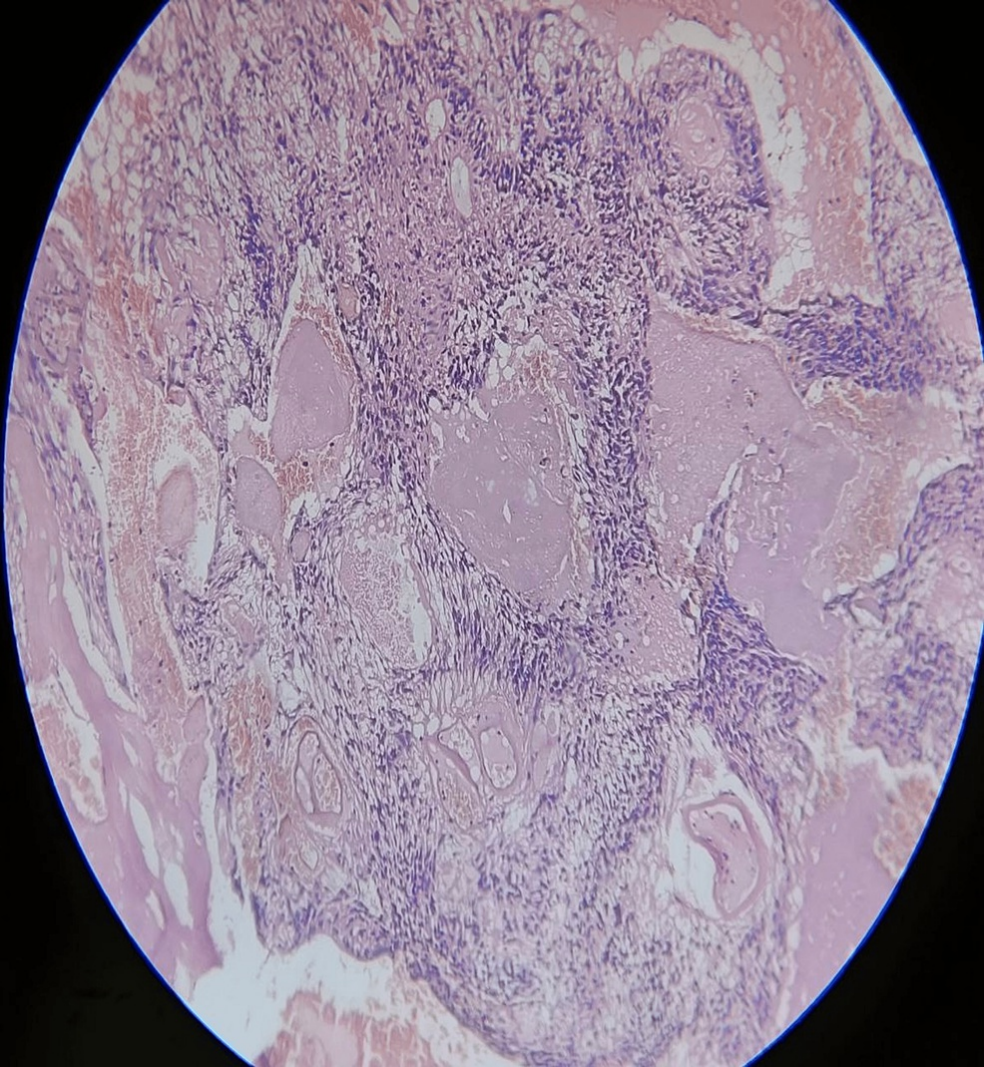
Postoperative histopathological section showing the atypical oval nuclei and pseudo rosettes around the vessels

**Figure 6:**
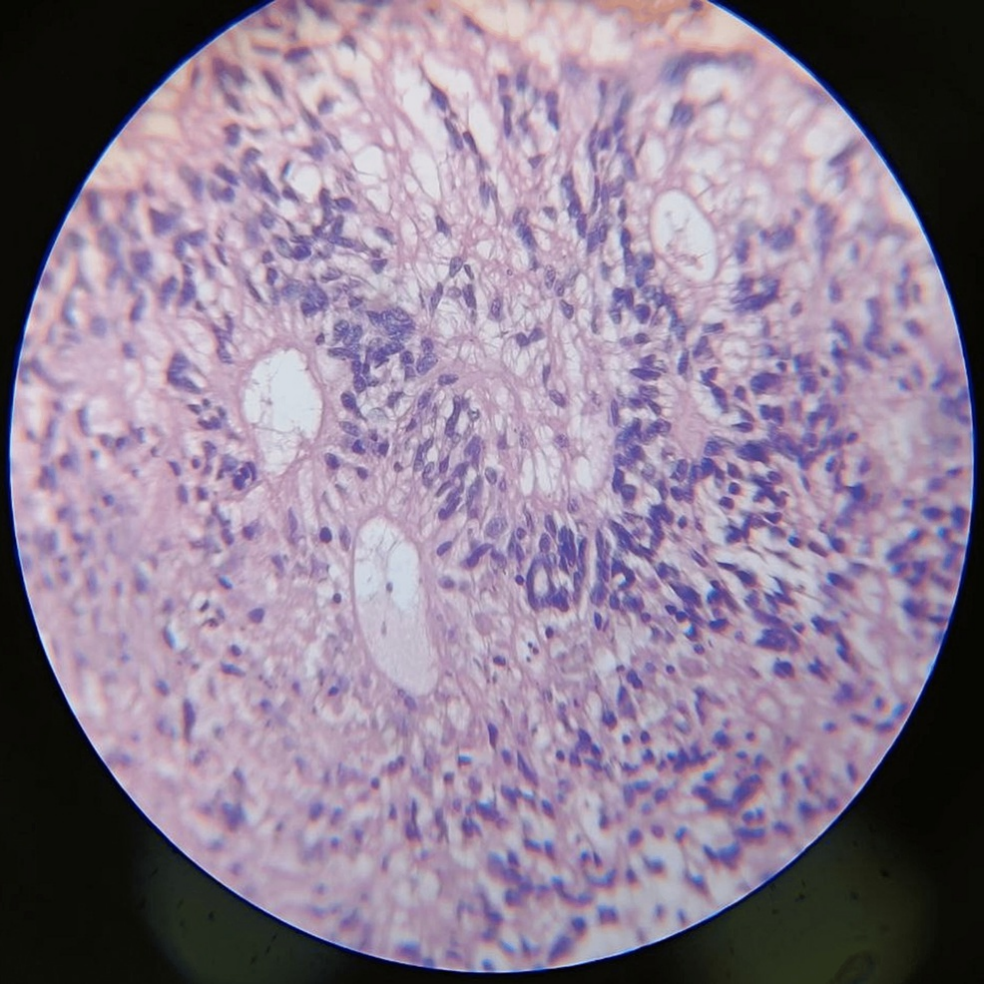
Histopathological section of ependymoma showing round to oval atypical nuclei of the tumor

**Figure 7:**
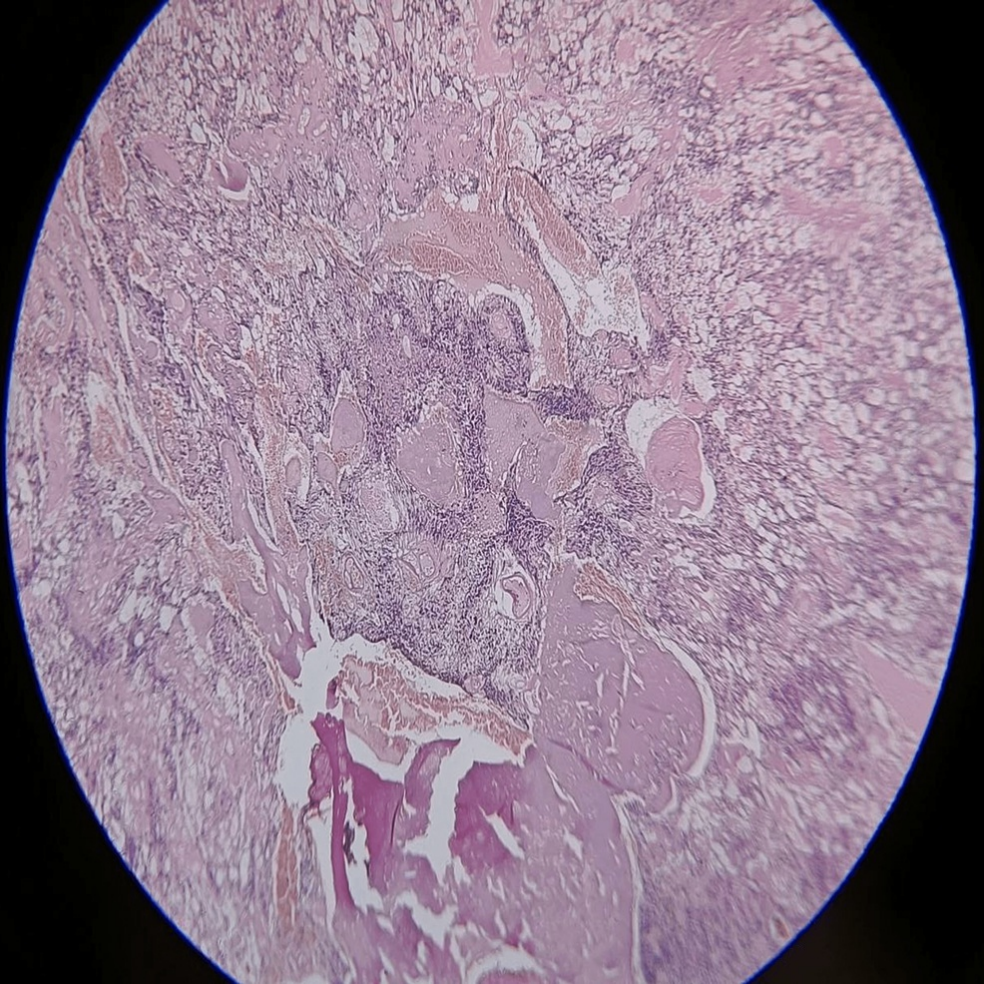
Postoperative histopathological section shows an ependymal canal with atypical nuclei surrounding it. Also, a hemorrhagic area can be spotted.

After the surgery, the patient's sample was sent for histopathological examination to confirm the diagnosis. The section shown in Figures 5-7 consists of perivascular pseudo rosettes. Tumor cells have round to oval nuclei with granular chromatin with considerable fibrillation in the background. Background also shows a few areas of hemorrhage and a few areas of necrosis.

Post-operative advice and follow-up

The power improved to grade III/V in the foot, ankle, knee flexors and extensors, hip flexors, extensors, abductors, adductors, and rotators of both sides. Urinary catheter clamping was started 48 hours after the operation. The patient started getting a bladder-filling sensation on day 5 of the surgery. She went home with the catheter. At the time of discharge, bladder training and catheter care were given to the patient and her attendants. The patient was discharged with the advice of back extension exercises, deep breathing exercises, vitamin B complex supplements, and plenty of fluid intake. 

On day 15 of surgery, on examination, the power had improved to grade IV/V in all lower limb muscles on both sides. The catheter was changed. After one month, the catheter was removed as the bladder and bowel control was restored and power was grade IV/V + in all the muscles of the lower limb on both sides.

## Discussion

Ependymomas are benign tumors of ependymal cells lining the central canal of the spinal cord and the brain parenchyma [2]. They can be neuroectodermal in origin. They are usually found in the pediatric population and adults over the age of 30 years. They are usually found in the sacrococcygeal region - cauda equina, filum terminale, and conus medullaris [1,2,4,9]. Ependymomas can be classified according to the appearance of the cells on the histopathological examination in various stages - GRADE I Myxopapillary Ependymoma; GRADE I Subependymomas; GRADE II Classic Ependymoma; GRADE III Anaplastic Ependymoma and RELA fusion-positive ependymomas [2,4,5].

The myxopapillary ependymomas show myxoid background with round to oval nuclei that form rosette-like structures around the vessels with some hemorrhagic focus and some collagen accumulation. Sub-ependymomas show hypocellular clusters of cells in an abundant glial matrix. Classic ependymoma shows a papillary pattern, clear cells regularly arranged in a true rosette; tanycytic is a rare variety with elongated spindle-shaped cells with eosinophilic fibrillary processes arranged in bundles. Anaplastic ependymomas show hypercellular, hyperchromatic cells in a pseudo-palisading pattern with microvascular proliferation without any rosettes. RELA fusion-positive ependymomas include sub-tentorial ependymomas in the pediatric and adult populations [2,4]. 

As far as the etiology is concerned, it varies with the age the tumor presents and the location of the tumor. The grade of the tumor also varies with the genetic aberrations. Few studies point towards the Warburg Effect, and other metabolic pathway alterations may lead to tumor growth. Upregulation of HIF1 alpha increased expression of *HOX* family genes, and other genetic aberrations may be analyzed for ependymomas. For example, spinal ependymomas are more related to the *NF2* mutation. Also, the *MDM2* overexpression or mutation may be responsible for the dysfunction of the *p53* gene, thus leading to the uncontrolled growth of the cells. The exact etiology is not yet clear, though [9].

Clinical features of myxopapillary ependymoma are very vague. They can range from lower back pain to any neurological symptoms due to compression or irritation of any nerve root. In this case, the patient had paraparesis and a positive Lasigue straight leg raising sign. It may also present with vertigo, any ophthalmic symptoms due to papilloedema (intracranial ependymoma), vomiting, etc. [4,9].

Differential diagnoses of myxopapillary ependymomas can be schwannoma, paraganglioma, fibrous meningioma, chordoma, giant cell tumor, or meningioma [6]. Treatment options include complete resection (which is Gold Standard treatment) and if a subtotal resection is performed, then re-operation to complete resection should be considered. Also, adjuvant radiotherapy has a role in selected cases. Recently, bevacizumab has been under trial to treat ependymomas as it has shown efficacy in treating neurofibromas [4,6]. In this case, the patient had undergone posterior decompression and tumor excision at the D12-L1 level for space-occupying lesions over D12-L1. Radiotherapy treatment is still not so useful as the tumor is slow growing and, the majority of the time, not invasive for adjacent structures. Although, postoperative radiotherapy can be advised to prevent long-term recurrence [2,6,9].

The prognosis depends upon the grade, location, and invasion of the tumor [1,4]. Also, the age of the patient determines the prognosis of the disease after surgery [1,4]. If the tumor perforates the capsule and CSF seeding has occurred, it is an alarming sign for tumor dissemination. Myxopapillary ependymomas have a better survival rate as compared to other varieties [1,4].

## Conclusions

This case report describes the rare spinal cord tumor, Myxopapillary Ependymoma (WHO grade I), that presented with lower back pain and paraplegia. This case is rare in this age group, and the tumor's location is different from the conventional one, which is expected to happen in the cauda equina and filum terminale. Treatment and prognosis depend upon the grade, location, and various patient factors.
